# Exosome and virus infection

**DOI:** 10.3389/fimmu.2023.1154217

**Published:** 2023-03-30

**Authors:** Yiqiu Peng, Yuxi Yang, Yingying Li, Tingjuan Shi, Yingyi Luan, Chenghong Yin

**Affiliations:** Department of Central Laboratory, Beijing Obstetrics and Gynecology Hospital, Beijing Maternal and Child Health Care Hospital, Capital Medical University, Beijing, China

**Keywords:** exosome, virus infection, HIV, HBV, HCV, SARS-CoV-2

## Abstract

Exosomes are messengers of intercellular communication in monolayer vesicles derived from cells. It affects the pathophysiological process of the body in various diseases, such as tumors, inflammation, and infection. It has been confirmed that exosomes are similar to viruses in biogenesis, and exosome cargo is widely involved in many viruses’ replication, transmission, and infection. Simultaneously, virus-associated exosomes can promote immune escape and activate the antiviral immune response of the body, which bidirectionally modulates the immune response. This review focuses on the role of exosomes in HIV, HBV, HCV, and SARS-CoV-2 infection and explores the prospects of exosome development. These insights may be translated into therapeutic measures for viral infections and reduce the disease burden.

## 1 Introduction

**Figure 1:**
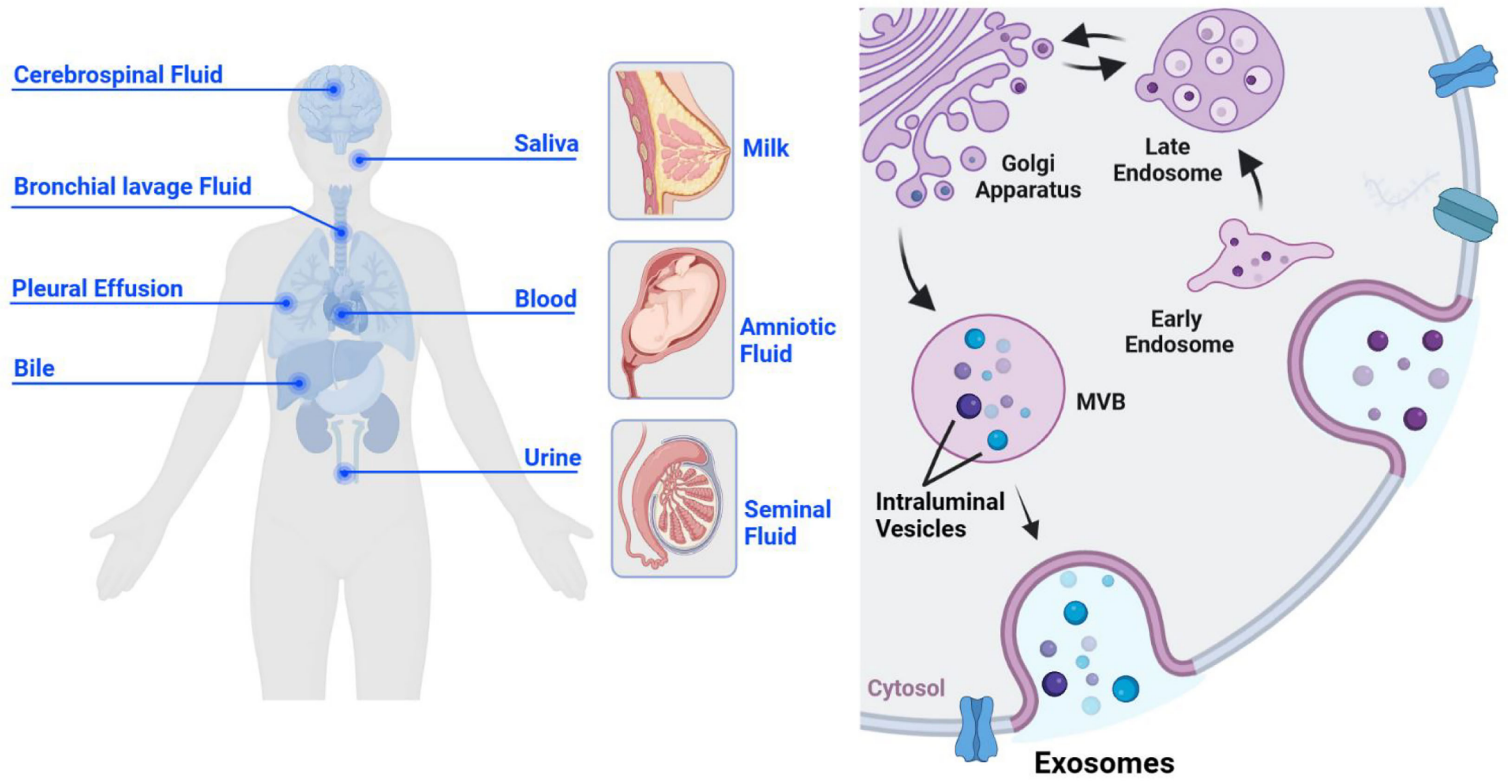
The origin and biogenesis of exosomes. Exosomes are derived from cells and are widely present in various body fluids, such as cerebrospinal fluid, saliva, bronchial lavage fluid, pleural effusion, blood, bile, urine, breast milk, amniotic fluid, and semen (left). Exosomes originate from the inward budding of the cell membrane, undergo early endosomes, late endosomes, and MVBS, and eventually form mature monolayer vesicles, which are secreted to the outside of the cell in the form of the exosome (right).

Exosomes are cell-derived extracellular vesicles with a single-layer membrane structure, approximately 30-150 nm in diameter. They serve as a communication bridge between cells and are significant in cellular remodeling contents. Exosomes are widely found in various body fluids, such as blood, urine, breast milk, semen, saliva, amniotic fluid, pleural effusion, ascites, bile, cerebrospinal fluid, and bronchial lavage fluid (1–7) (**Figure 1**). Further, they are widely involved in the pathophysiological process of tumors, inflammation, immunity, and infection (8–11). As early as 1983, Pan found that sheep reticulocytes released a kind of vesicle containing transferrin receptors *in vitro* (12), which could be harvested by high-speed centrifugation. Until 1987, this vesicle was named exosome, which provided a selective pathway for membrane protein loss for erythrocyte maturation (13). Since then, studies on exosomes have flourished.

Viruses are tiny, simple, non-cellular organisms that must parasitize inside living cells to proliferate. More and more studies have found a close relationship between viruses and exosomes. For example, retroviruses can borrow vesicle production mechanisms to promote budding (14), and hepatitis viruses can use exosomes for cell-to-cell transmission (15–17). In addition, exosomes establish a long-term connection between virus and host by activating innate immunity and antiviral immune response. This paper reviews the origin and contents of exosomes emphasizing the role of exosomes in human immunodeficiency virus (HIV), hepatitis B virus (HBV), hepatitis C virus (HCV) and SARS-CoV-2 infection, as well as in the body’s immune response.

## 2 Biogenesis and cargos

The production process of exosomes is complex and elaborate, regulated by multiple molecules. Unlike ectosomes, exosomes originate from the inward sprouting of the cell membrane (18, 19), forming the early endosomes. All kinds of intracellular goods are exchanged with the early endosome material and recycled to promote the maturation of late endosomes (20). Late endosomes can eventually form multivesicular bodies (MVBs), which isolate and sort their cargos through intraluminal vesicles (ILVs). It is released into the extracellular space as exosomes (21) (**Figure 1**). The endosomal sorting complex required for transport (ESCRT) plays an important regulatory role throughout the process of exosomes generation and transport to the extracellular space. ESCRT-0 interacts with phosphatidylinositol 3-phosphate (PtdIns3P), a specific receptor on the outer surface of early endosome, *via* the ubiquitin binding site hepatocyte growth factor-regulated tyrosine kinase substrate (HRS) FYVE domain (22), budding inward in a “reverse budding” manner, and selectively wraps part of the cytoplasm to initiate the MVBs pathway. Initial membrane deformation and cargo sorting are completed with the assistance of ESCRT-I and II (23), and membrane deformation and splitting occur under the shearing operation of ESCRT-III to form MVBs (24, 25). Further, there are several studies proposing an ESCRT independent manner of exosome formation. Wei (26) found that high levels of RAB31 can prevent MVBs from fusing with lysosomes and drive epidermal growth factor receptor (EGFR) into MVBs, so that ILVs can be secreted as exosomes. The CD63 and the sphingolipid ceramide have also been identified as important effectors of the ESCRT-independent MVBs generation mechanism (27, 28).

In the early stage, we considered exosomes to be cell-derived debris. With the deepening of understanding, it is realized that exosomes are of great significance in immune regulation and intercellular communication. The function of exosomes hinges on the diversity of the intact membrane structure and contents. According to the biogenesis mechanism of exosomes, their single membrane vesicles contain a variety of cell-derived lipids. However, interestingly phosphatidylcholine (PC) is reduced in exosomes compared to cells, and cholesterol, sphingomyelin, glycosphingolipids, and phosphatidylserine (PS) are enriched 2-3 times in exosomes (29). The extravasation of PS to the outside of the cell membrane is often used as a marker of apoptosis. However, some studies have shown that PS can be detected in the outer membrane of some exosomes by Annexin-V binding assay. This lipid component translocation may be related to host cell type and secretory state (29, 30). Exosomes contain various proteins, which are crucial executors of life process, including evolutionarily conserved Rab proteins, actin/myosin, tubulin, and annexin, as well as proteins necessary for exosome production and function, such as MVB synthesis-associated proteins (e.g., Alix, TSG101), heat shock proteins (HSC70, HSC90), and tetraspanin proteins (CD9, CD82, CD81, CD63) (31, 32). Among them, CD9, CD63, and CD81 are the most commonly used specific markers (33, 34). Exosomes originate from different cells containing specific proteins. For example, exosomes released by virus-infected cells contain viral protein particles associated with virus transmission. Exosomes secreted by dendritic cells (DCs) contain functional major histocompatibility complex (MHC) and antigen peptide complexes, which can activate cytotoxic T-lymphocyte (CTL) in mice to generate anti-tumor immunity (35). In addition to lipids and proteins, exosomes contain a variety of nucleic acids. In 2007, exosome-mediated mRNAs and microRNAs (miRNAs) were first discovered to be transferred between cells, which became a new mechanism of genetic exchange (36). Subsequently, gene silencing caused by the delivery of miRNAs to target cells through exosomes has gradually become a research direction for multiple diseases (37, 38). In 2014, Thakur found the presence of double-stranded DNA in exosomes for the first time, which could represent the whole genomic DNA and had the function of identifying mutations in parental tumor cells (39), adding dsDNA to a list of other nucleic acids found in exosomes.

## 3 Exosomes in virus infection

Exosomes affect the viral infection process in two aspects by adjusting their contents as well as the immune status of the body. On the one hand, it promotes virus replication, transmission, and infection and down-regulates antiviral immunity. On the other hand, it limits virus infection and potentiates antiviral immunity. Based on these two directions, we will summarize the “love and hate “ between exosomes and the following viral infections.

### 3.1 Exosomes in HIV

The generation of exosomes is similar to that of RNA viruses, especially retroviruses. HIV is the first RNA virus to be used for exosome research, and it is also one of the most intensively studied viruses. In 2003, Gould proposed “the Trojan exosome hypothesis.” The hypothesis holds that retroviruses use preexisting exosome biogenetic pathways to form infectious particles and share protein targeting and biogenetic pathways with exosome, as well as preexisting exosome uptake pathways to form receptor-independent and Env-independent infection ways to promote viral infection (40). Negative factor (Nef) is an HIV-1 accessory protein essential for viral replication and down-regulation of CD4, MHC-I (41, 42). CD4 is the receptor molecule required for HIV-1 infection and CD4^+^ T cell count is considered an important component of HIV disease management and the gold standard for assessing disease progression (43, 44). CD4 on exosomes derived from CD4^+^ T cells can reduce HIV-1 infection, while Nef in infected T cells can decrease CD4 expression in exosomes, effectively enhancing HIV-1 infection (45). Exosomes containing Nef can promote the secretion of itself, increase intracellular MVBs and induce apoptosis of CD4^+^ T cells, which contribute to the depletion of CD4^+^ T cells in the pathogenesis of AIDS (46, 47). Autophagy is a defense mechanism against HIV, and Nef bypasses autophagy-mediated restriction by promoting ubiquitination of BCL2, an autophagy inhibitor, through the Parkin/PRKN pathway (48). C-C chemokine receptor type 5 (CCR5), a coreceptor of HIV-1, is also detected in exosomes secreted by HIV-1-infected cells, and HIV-1 may transfer receptor molecules through exosomes to alter the expression of viral receptors on the cell surface, which is conducive to HIV-1 infection of tissues with endogenous CCR5 expression loss (49), and the differential expression of CCR5 molecule on the exosome membrane may help to distinguish HIV patients with different progression types (50). Group-specific antigen (Gag) is also contained in exosomes released by HIV-infected cells. Gag and its cleavage fragments are critical for the assembly and infectivity establishment of virions (51), while it can interact with tetraspanins, such as CD63 and CD81, to aid virion egress (52). The most abundant HIV-1 derived miRNA is trans-activation response element (TAR) miRNA, which mainly exists in the exosomes of HIV-infected cells. Exosome TAR RNA down-regulates apoptosis of recipient cells by decreasing Bim and Cdk9 proteins and finally enhances the infection process of HIV (53). Exosomes derived from HIV-infected cells also contain Nef mRNA, which can be delivered to human neuroblastoma cells and translated into Nef protein, leading to HIV-associated neurocognitive disorders (54). In brief, HIV widely mobilizes accessory protein, receptors and nucleic acids to promote the occurrence of virus infection.

Although exosome can promote the spread of HIV-1 and escape host immunity, it also plays an important role in HIV antiviral immunity. Exosomes in healthy human semen block the transmission of HIV-1 from vaginal epithelial cells to target cells and inhibit HIV-1 from crossing the vaginal epithelial barrier in a trans-well model *in vitro* (55). More importantly, after the internalization of seminal exosomes, they exert an antiviral response by blocking the activity of HIV-1 reverse transcriptase and reduce the replication of the viral complex, which has been demonstrated in mice (56). Seminal exosomes inhibit the recruitment of NF-κB, Sp1, and RNA Pol II to the promoter region of HIV-1 5’ long terminal repeats (LTR). It also inhibits the binding and recruitment of HIV-1 trans-activating protein (Tat) to HIV-1 LTR, thereby silencing HIV-1 RNA expression (57). Human breast milk exosomes exert protective effects against vertical transmission of HIV-1 by competing with HIV-1 to bind DC-specific ICAM-grabbing non-integrin (DC-SIGN) on monocyte-derived dendritic cells (58). Exosomes in the culture supernatant of HBMECS (human brain microvascular endothelial cells) activated by TLR3 contain a variety of antiviral factors that can be delivered to macrophages, which may be a defense mechanism against HIV invasion in the central nervous system (59). A similar pattern has been found in the gastrointestinal tract where Tlr3-activated intestinal epithelial cells released exosomes containing anti-HIV factors. Treatment of macrophages with supernatants containing these exosomes inhibited HIV replication and induced the expression of antiviral interferon (IFN)-stimulated genes (ISGs) and cellular HIV restriction factors (Tetherin and APOBEC3G/3F) in HIV-Infected macrophages (60). These results suggest that the anti-HIV effect of exosomes may be an essential component of innate immunity.

### 3.2 Exosomes in HBV

By 2015, there were about 257-270 million HBV carriers worldwide, the prevalence of chronic hepatitis B virus infection was approximately 3.5% (61), and about 71 million people were infected with HCV, accounting for 1% of the population (62). Faced with such a vast health economic burden, the World Health Organization (WHO) adopted the first global strategy on viral hepatitis in 2016, calling for the elimination of viral hepatitis by 2030. Therefore, many countries and regions have actively responded to this global strategic goal and formulated corresponding plans. It is urgent to explore the pathological mechanism and treatment of viral hepatitis. Hepatitis virus is divided into five types, HAV, HBV, HCV, HDV, and HEV. Among them, HAV and HEV are self-limited diseases mainly transmitted by the fecal-oral route (63–65), which are not easy to develop into chronic hepatitis. HDV is a defective virus that needs to be assisted by HBV to replicate and proliferate (66, 67). However, there are many patients infected with HBV and HCV, and the course of the disease is easy to extend and develop into a chronic infection. Some patients can progress to liver cirrhosis and liver cancer, which not only dramatically affects the life and health of patients, but also seriously increases the global health and economic burden. Therefore, our discussion will focus on HBV and HCV.

HBV is an enveloped DNA virus with a 3.2 kb partially double-stranded DNA genome that encodes four transcripts, 3.5 kb mRNA, 2.4 kb mRNA, 2.1 kb mRNA, and 0.7 kb mRNA. There are four major open reading frames (ORFs): S, P, C, and X, which encode hepatitis B surface antigen (HBsAg), DNA polymerase (DNA pol), core protein (HBcAg and HBeAg), and X protein (HBx), respectively. Exosomes derived from the peripheral blood of CHB patients contain abundant viral components, including HBV-DNA, HBV-RNA, HBsAg, and HBeAg, which can induce active infection in human hepatocytes. It can also be transported to NK cells to inhibit NF-kB and p38 MAPK signaling pathways, leading to their dysfunction that destroys the innate immune response and promotes the process of HBV infection (16). Recent studies have found that exosomes released by HBV-infected cells contain intact viral particles (68), which provided strong evidence for the transmission of HBV through the exosome pathway. Although the detailed mechanism of HBV entry into exosomes has not been fully elucidated, HBV may have some similarities with the biological mechanism of exosomes. HBV envelope protein was co-localized with MVB proteins AlP1/ALIX and VPS4B in human hepatocellular carcinoma cells. AlP1 or VPS4B mutations can inhibit virus production and release (69). Exosomes can bind with ESCRT-related proteins and be transported through ceramide-dependent pathways to achieve the purpose of virus transmission (70). HBsAb is an essential immune protein against HBV infection. In the presence of HBcAg^+^ CD81^+^ extracellular vesicles, the protective effect of HBsAb is significantly reduced, suggesting that CD81^+^ extracellular vesicles are involved in the immune escape of HBV (71). Thus, exosomes can effectively transmit HBV by providing a biogenetic mechanism, acting as an immune barrier, and enhancing the ability of HBV replication.

Exosomes not only contain HBV-related viral components but also derive a large number of non-coding RNA. By examining extracellular vesicles secreted by HBV-infected human hepatocytes, Enomoto found that miR-21, miR-192, miR-215, miR-221, and miR-222 directly targeted multiple sequences in the 3’UTR of human IL-21 mRNA. Moreover, it inhibits the expression of IL-21 in human T cells and ultimately weakens the body’s antiviral ability (72). HBV-miR-3 is highly expressed in peripheral blood exosomes of patients with hepatitis B, and it targets the 3.5-kb HBV transcript to inhibit HBV replication (73). However, miR-3 also exerts antiviral immune effects. It has been suggested that the down-regulation of SocS5 by HBV-miR-3 in hepatocytes can activate JAK/STAT signaling pathway and enhance the IFN-induced anti-HBV effect. In addition, HBV-miR-3 in exosomes can promote the M1 polarization of macrophages and the secretion of IL-6 (74). MiR-122 is considered a biomarker of various liver injuries. HBV infection is associated with increased expression of miR-122, the serum miR-122 level in CHB patients is significantly higher than in healthy people. In contrast, the miR-122 decreases in patients with advanced liver fibrosis. Therefore, miR-122 can reflect the degree of liver inflammation and is related to the disease progression of HBV infection (75).

Exosomes are involved in the release and transmission of HBV and interact with immune cells to regulate the immune response. Extracellular vesicles secreted by HBV-infected cells are endocytosed by monocytes which play an immunosuppressive role by up-regulating PD-L1 and inhibiting the expression of CD69 (76). The physiological role of PD-1 is to ensure the homeostasis of T cells by limiting its activation and proliferation. The consumption and inactivation of T cells in CHB patients may be caused by the increased PD-L1 expression in monocytes induced by HBV-related exosomes (77, 78). Kouwaki (79) found HBV-infected hepatocytes can release exosomes containing viral nucleic acids to stimulate the expression of NKG2D ligand in macrophages through MyD88, TICAM-1 and MAVS-dependent pathways in a tree shrew animal model of HBV infection. The expression of NKG2D ligands in macrophages activates NK cells, which produce IFNγ in the early stage of viral infection and promote the degradation of HBV nucleic acid in the cytoplasm, thus playing an antiviral role. Other studies have suggested that the uptake of HBV-positive exosomes by NK cells will impair the function of NK cells, including interferon (IFN) production, killing activity, NK cell proliferation, and survival, and response to poly (l: C) stimulation (16). All these studies have clarified the critical role of exosomes in the immune response related to HBV infection, which is expected to become a potential target for antiviral therapy. In general, the role of exosomes in HBV infection varies with the goods they carry. When the contents are viral proteins, nucleic acids, and other substances, exosomes promote HBV diffusion. When the contents are anti-HBV factors, exosomes have the effect of inhibiting viral activity and antiviral infection. At the same time, the cargos also act as a ligand of immune cells to activate antiviral immunity and play a positive role.

### 3.3 Exosomes in HCV

Hepatitis C virus (HCV) is an enveloped hepacivirus that carries a single-stranded positive-sense RNA genome (80) and encodes the components of viral particles after entering hepatocytes. It includes three structural proteins (core, E1 and E2) and seven non-structural proteins (p7, NS2, NS3, NS4A, NS4B, NS5A and NS5B) (81). HCV infection has become a global health problem because it mainly causes chronic inflammation and necrosis of the liver, liver fibrosis, cirrhosis, and even hepatocellular carcinoma. HCV can infect cells in a receptor-mediated manner, and its envelope protein E2 can interact with the CD81 receptor on the cell surface, allowing HCV to enter and infect cells (82). However, the detection rate of exosomes-associated HCV in the plasma of HCV-infected patients is higher than non-exosome HCV, suggesting that the non-receptor-dependent mode of HCV infection has important research value (83). HCV-infected hepatocytes can secrete exosomes carrying HCV RNA and protein and transmit the contents to other hepatocytes. Due to the protective effect of exosomes, this transmission route can attenuate the effect of antiviral antibodies to a certain extent (84), which may provide an effective way for the HCV to escape immunity (85). Bukong found that the exosomes of HCV-infected hepatocytes contain Ago2 protein, HSP 90, and miRNA-122, which can stabilize HCV RNA and promote HCV transmission (86). HCV envelope glycoprotein E2 not only exists on the virus surface but also can be released through the exosome pathway. Under the regulation of syntenin, the secretion of exosomes coated with E2 protein increases and makes the HCV difficult to be neutralized by specific antibodies and serum of patients in the chronic phase (87) so that HCV remains infectious. In addition, the E2 protein can up-regulate miR-490 of mast cells in the tumor microenvironment, which is transported to liver cancer cells by exosomes to inhibit the ERK1/2 signaling pathway, and ultimately inhibit liver cancer metastasis, providing a treatment idea for liver cancer induced by HCV infection (88). Currently, miRNAs play an important role in the pathogenesis of hepatitis C (89). MiR-122 is a biomarker of liver injury and an essential regulator of HCV replication (90). Jiao found that under the treatment of PEG-IFN plus ribavirin, the average level of serum miR-122 in the sustained virological response (SVR) group was higher than that in the non-response (NR) group, and the level of exosomal miR-122 in the SVR group was also higher than that in the NR group. It indicates that miR-122 in serum and exosomes may be related to the therapeutic effect of viral hepatitis (91). Circulating miR-122, miR-199a, and miR-16 can be used to early detect HCV infection complicated with hepatocellular carcinoma (92). HCV-induced miR-192 can be transferred from hepatocytes to hepatic stellate cells (HSCs) by exosomes and up-regulate the fibrosis markers in HSC through TGF-β1 to accelerate the formation of hepatic fibrosis. At the same time, HSC differentiation can be reversed after anti-miR-192 treatment (93). HCV-EXOs carrying miR-19a can also target HSC to promote fibrosis formation and activate the STAT3-TGF-β signaling pathway through SoCS3 in HSC. The signaling pathway ultimately enhances fibrosis marker genes (94). miR-155 participates in various inflammatory responses and is a key regulator of cellular function in both innate and acquired immunity. The expression of miR-155 is up-regulated in HCV-infected liver tissue. Overexpression of miR-155 can inhibit hepatocyte apoptosis, promoting proliferation and tumorigenesis (95). MiR-155 derived from B cells can inhibit HCV replication in hepatocytes through exosome transmission. Rituximab can not only induce B cell depletion but also affect the production of intracellular miR-155 and the delivery of miR-155 in exosomes, thereby enhancing HCV activity in hepatocytes (96). Therefore, a variety of miRNAs in exosomes regulate the progression of HCV infection, which has the potential value of developing into antiviral drugs and assisting in the judgment of disease progression. In terms of immunity, DCs from patients with persistent HCV infection still exhibit normal phagocytic activity, typical class I, class II HLA expression, and regular cytokine production (97, 98), indicating that DCs still exert antiviral immunity during persistent HCV infection. Then CD8+T cells are stimulated by exosome-activated DCs to play a joint role in cellular immunity (99). In the supernatant of HCV-infected hepatocytes, exosomes containing HCV RNA are the source of TLR3-mediated DC maturation (100). Another study found that exosomes containing HCV dsRNA intermediates can reduce the activation of TLR3, which is conducive to the persistent infection of the virus (101). Activated macrophages transfer miRNA29 through exosomes to inhibit HCV replication in Huh7 cells, and either exosomal inhibition or miRNA-29 inhibition abolished this antiviral effect (102). In the end, the game between HCV infection and antiviral immunity determines the outcome of the disease. In the supernatant of HCV-infected hepatocytes, exosomes containing HCV RNA is the source of TLR3-mediated DC maturation (100). Another study found that exosomes containing HCV dsRNA intermediates can reduce the activation of TLR3, which is conducive to the persistent infection of the virus (101). Activated macrophages transfer miRNA29 through exosomes to inhibit HCV replication in Huh7 cells, and either exosome or miRNA-29 inhibition abolished this antiviral effect (102). Ultimately, the game between HCV infection and antiviral immunity determines the outcome of the disease.

### 3.4 Exosomes in SARS-CoV-2

Corona Virus Disease 2019 (COVID-19) is an infectious disease caused by SARS-CoV-2 infection, which broke out at the end of 2019 and spread rapidly around the world (103). As of December 23, 2022, 651,918,402 confirmed cases and 6,656,601 deaths have been reported to WHO. The symptoms of patients vary from common influenza to severe pneumonia, and the mechanism research of pathological process involves acute respiratory distress syndrome, inflammatory factor storm, multiple organ dysfunction and coagulation dysfunction (104, 105). At present, a variety of vaccines have been used on a large scale to strengthen the immune defense line of the population. However, clinical antiviral treatment is limited, severe patients are in urgent need of safe and effective intervening measures.

SARS-CoV-2 relies on spike protein (S protein) binding to angiotensin-converting enzyme 2 (ACE2) on the cell membrane. With cleavage by transmembrane protease, serine 2 (TMPRSS2), on the cell surface, the fusion pore is formed to provide an infection channel for the viral genome entry into the cell (106). Some studies have shown that ACE2-expressing extracellular vesicles (evACE2) are increased in the plasma of patients with COVID-19. These vesicles have specific exosome markers, neutralize SARS-CoV-2 by competitive binding to ACE2, and have the same or higher inhibitory effect on mutant strains. At the same time, it can reduce the mortality of SARS-COV-2 infection in hACE2 mice (107), providing a new treatment strategy for COVID-19. IFN α/β treatment increases the expression of exosomal hACE2, and exosomal hACE2 specifically blocks viral entry into target cells, inhibiting the replication of SARS-CoV-2 *in vitro* and ex vivo (108). This pretreatment modality has a particular strengthening effect on improving the antiviral effect of exosomes. Current studies have shown that a SARS-CoV-2 intranasal vaccine based on extracellular vesicles of Salmonella typhimurium can induce neutralizing antibodies against the wild type and Delta variant strain, alleviate lung lesions and improve weight loss, highlighting the value of extracellular vesicles in SARS-CoV-2 vaccines (109). Barberis detected SARS-CoV-2 RNA in the exosome of COVID-19 patients for the first time by using reverse transcription-droplet digital polymerase chain reaction. At the same time, the proteomic analysis found that the exosomal cargo was related to immune response, inflammation, coagulation pathway, and pathology-related clinical indicators. The researchers believed exosome was expected to become a biomarker of COVID-19 (110). In addition, the composition of serum-derived exosomes from COVID-19 patients was in connection with the disease severity. Vesicles from patients with the mild disease could modulate antigen-specific CD4 T cell responses, and the exosomal proteome was associated with the normal function of the immune system. However, the proteome of exosomes from patients with severe disease was associated with chronic inflammation (111). Therefore, the in-depth analysis of exosome components helps us to understand the pathological state with different degrees of disease. Based on the potential value of exosomes in the diagnosis and treatment of COVID-19, 14 clinical trials of exosomes and COVID-19 have been registered (**Table 1**) (ClinicalTrials.gov: https://clinicaltrials.gov/, Chinese Clinical Trial Registry: http://www.chictr.org.cn/), of which mesenchymal stem cell-derived exosomes is the most attractive target for research. A prospective study involving 24 severe COVID-19 patients found that a single-dose intravenous treatment of bone marrow mesenchymal cell-derived exosomes significantly improved clinical status and oxygenation while reducing absolute neutrophil count and acute-phase reactants such as C-reactive protein. It suggests that exosome derived from allogeneic bone marrow mesenchymal stem cells is a safe and effective drug candidate for treating severe COVID-19 (112). As an important carrier of non-coding RNA, exosomes can intervene in the expression of target genes by delivering miRNAs. Some studies (113, 114) predict the interaction between non-coding RNA and viral transcripts and the targeting sites by computer, looking for miRNAs with potential therapeutic value. Our understanding of SARS-CoV-2 is still limited, but with the deepening of various studies and combing the experience of other coronaviruses, we will have a more comprehensive understanding of the pathological mechanism of COVID-19 and develop more scientific prevention and treatment measures.

**Table 1:** Clinical trials of exosomes and COVID-19.

Registration number	The source of exosome	Number of people	Title	Intervening measure
1 NCT04276987	mesenchymal stem cells	24	A Pilot Clinical Study on Aerosol Inhalation of the Exosomes Derived From Allogenic Adipose Mesenchymal Stem Cells in the Treatment of SeverePatients With Novel Coronavirus Pneumonia	5 times aerosol inhalation of MSCs-derived exosomes (2.0x10^8^ nano vesicles/3 ml at Day 1- Day 5.
2 NCT04969172	human embryonic kidney T-REx^TM^ -293 cells	155	A Phase II Randomized, Double-blind, Placebo-controlled Study to Evaluate the Safety and Efficacy of Exosomes Overexpressing CD24 to Prevent Clinical Deterioration in Patients With Moderate or Severe COVID-19 Infection	The exosomes are diluted in 4ml normal saline for inhalation, administered once daily (QD) for 5 days.
3 NCT05216562	mesenchymal stem cells	60	Efficacy and Safety of EXOSOME-MSC (Mesenchymal Stem Cell-Derived Exosomes) Therapy to Reduce Hyper-inflammation In Moderate COVID-19 (2019-New Corona Virus Disease) Patients	The exosomes are injected to participants via intravenous route twice, in day-1 and day-7 of 14 days of study participation.
4 NCT04902183	–	90	A Phase II Randomized, Single-blind Dose Study to Evaluate the Safety and Efficacy of Exosomes Overexpressing CD24 in 10^9Dose Versus 10^10 Dose, for the Prevention of Clinical Deterioration inhalation via mouthpiece nebulization, administered in Patients With Moderate or Severe COVID-19	The exosomes are diluted in normal saline for inhalation via mouthpiece nebulizer, administered once daily (QD) for 5 days
5 NCT04602442	mesenchymal stem cells	90	The Extended Protocol of Evaluation of Safety and Efficiency of Method of Exosome Inhalation in COVID-19 Associated Two-Sided Pneumonia	Twice a day during 10 days inhalation of 3 ml special solution contained 0.5-2x10^^^10 of nanoparticles (exosomes).
6 NCT05191381	mesenchymal stem cells	40	Immune Modulation by Stem Cell Derived Exosomes in Critically III COVID-19	–
7 NCT04798716	mesenchymal stem cells	55	Mesenchymal Stem Cell Exosomes for the Treatment of COVID-19 Positive Patients With Acute Respiratory Distress Syndrome and/or Novel Coronavirus Pneumonia	MSC-exosomes delivered intravenously every other day on an escalating dose.
8 NCT04747574	human embryonic kidney T-REx^TM^ -293 cells	35	A Phase I Feasibility Study to Evaluate the Safety of CD24-Exosomesin Patients With Moderate/Severe COVID-19 Infection	EXO-CD24-exosomes aerosolized in normal saline for inhalation via a standard hospital-grade inhalation device, QD for 5 days.
9 NCT04389385	COVID-19 specific T Cell (CSTC)	60	Aerosol Inhalation of the Exosomes Derived From Allogenic COVID-19 T Cell in the Treatment of Early Stage Novel Coronavirus Pneumonia	Inhaler CSTC-Exo treatment are applied daily x 5 times (2.0x10^8^ nano vesicle/ 3 ml; on day 1 to day 5)
10 NCT05387278	placental	20	Safety and Effectiveness of Placental Derived Exosomes and Umbilical Cord Mesenchymal Stem Cells in Moderate to Severe Acute Respiratory Distress Syndrome (ARDS) Associated With the Novel Corona Virus Infection (COVID-19)	The treatment consists of administration of WJ-Pure^TM^ and EV-Pure^TM^ plus standard care for 5 days.
11 NCT04491240	mesenchymal stem cells	30	The Protocol of Evaluation of Safety and Efficiency of Method of Exosome Inhalation in SARSCoV-2 Associated Two-Sided Pneumonia	Twice a day during 10 days inhalation of 3 ml special solution contained 0.5-2x10^^^10 of nanoparticles (exosomes).
12 ChiCTR200003048490	–	90	HUMSCs and Exosomes Treating Patients with Lung Injury following Novel Coronavirus Pneumonia (COVID-19)	HUMSCs: intravenous infusion, 5 x 10^7^ cells / time, 1 time / week, 2 times / course, a total of 2 courses; Exosomes: intravenous administration, 180mg/ time, 1 time / day, 7 days / course, 2 courses in total.
13 ChiCTR2000030261	mesenchymal stem cells	26	A study for the key technology of mesenchymal stem cells exosomes atomization in the treatment of novel coronavirus pneumonia (COVID-19)	Aerosol inhalation of exosomes.
14 ChiCTR2000029982	peripheral blood	150	Prediction of prognosis and antiviral effect of patients with neocoronavirus infected pneumonia based on Peripheral blood exosomal metabolomics analysis and radiomics in CT of lung and its mechanism	–

-, unknown.

## 4 Prospect

**Figure 2:**
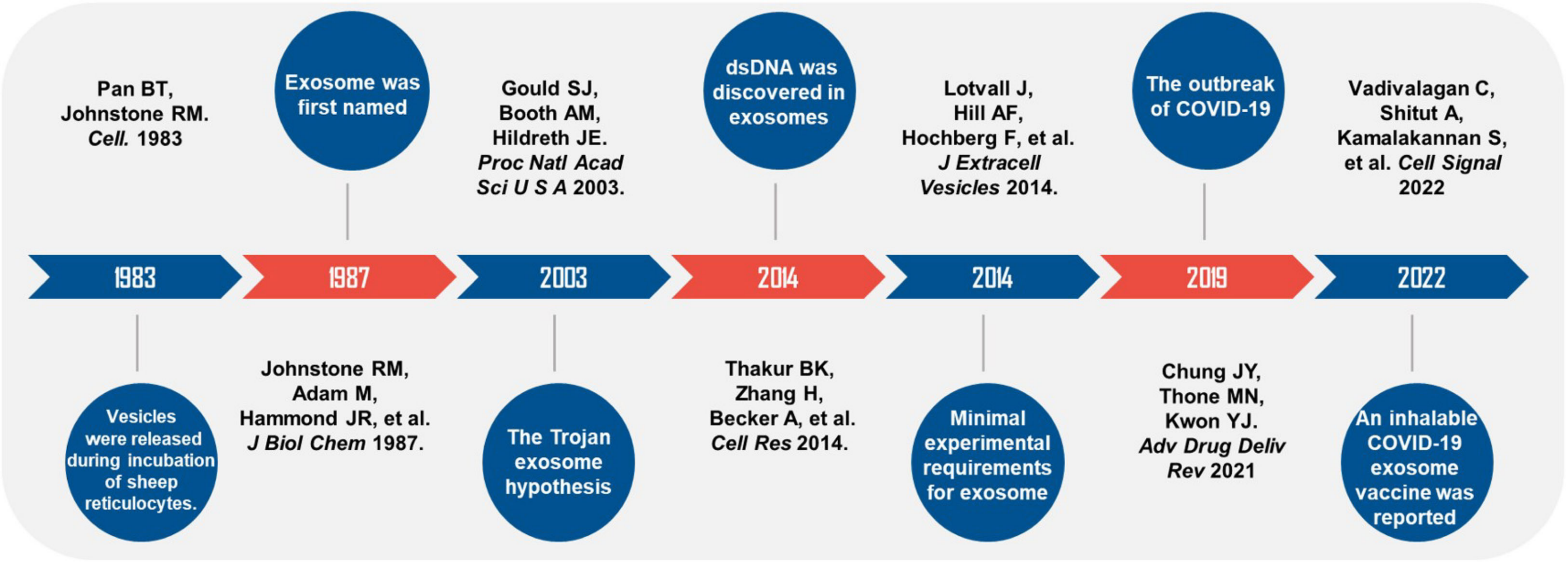
Timeline of studies on exosomes and viral infections. Black handwriting shows the author and the journal. Major events were described in white handwriting.

It has been 40 years since the exosome was discovered and named. During these four decades, scientists explored the relationship between the pathogenesis of viruses and exosomes and proposed “the Trojan exosome hypothesis”. Until COVID-19 swept the world in 2019, exosome vaccines were rapidly put into clinical studies to provide safe and effective preventive measures for epidemic prevention and control. (**Figure 2**). Scientists have extensively explored the exosome’s origin, the types of contents, and the interrelationships of exosomes with many diseases. With the development of multiple disciplines, clinical medicine, pharmacy, and engineering have all invested great interest in exosome. It has also shown unique charm in many fields, such as cancer, inflammation, infection, and aging. Exosomes carry the characteristics of the source cells and ensure the stable sequestration of cargo in vesicles from degradation. Therefore, exosomes can be used as liquid biopsy tools (5), especially for the early detection and stratification of tumors. Urine detection based on three exosomal RNA transcripts has been validated in the risk prediction of prostate cancer in 50,000 male patients and entered the early detection guideline (115). MicroRNAs carried in exosomes provide a unique perspective on the diagnosis and prognosis of breast, lung, and colorectal cancer (116) and are considered promising biomarkers. Regarding treatment, exosomes are derived from cells and have good histocompatibility and stability, which are ideal carriers for many drugs. Studies have shown that curcumin can be delivered to activated myeloid cells *in vivo* through exosomes to suppress the inflammatory response, which elevates the drug concentration in the blood and limits the side effects caused by off-target effects, providing targeted therapy for inflammatory diseases (117). In addition to this, exosomes have great potential in the development of viral and anti-tumor vaccines. Kuate found that the combination of the priming effect with exosome vaccine containing S protein and booster immunization with adenovirus vector could produce neutralizing antibodies with a higher titer than that of serum in convalescent SARS patients (118). The existing SARS-CoV-2 mRNA vaccines require cold chain preservation and induce a systemic immune response by intramuscular injection. Some researchers have combined lung-derived exosomes with recombinant SARS-Cov-2 receptor binding domain (RBD) to form an inhaled COVID-19 vaccine, which can enhance the retention of RBD in the respiratory tract and lung parenchyma. It can reduce severe pneumonia induced by the live vaccine and can be stably stored at room temperature for three months, which solves the temperature problem during vaccine storage and distribution (119). Dendritic cells are potent antigen-presenting cells that activate T cells, B cells, NK cells, and other immune cells, participating in anti-tumor immune responses in various ways. It has been proved that exosome vaccines derived from dendritic cells are safe and feasible in treating non-small cell lung cancer and metastatic melanoma (120, 121), which provides a breakthrough for tumor immunotherapy. In 2014, the International Society for Extracellular Vesicles proposed the minimum experimental standards for extracellular vesicles and their functions (122).

However, we still need to solve a series of problems on the road of exosomes from the laboratory to large-scale production and application. For example, the diameters of exosomes and viral particles are similar, so it is difficult to exclude the contamination of viral particles by using differential ultracentrifugation alone. It was found that the combined application of Polyethylene Glycol can achieve the purpose of purifying exosomes by isolating the virus (123). Other commonly used separation methods include ultrafiltration, immunoaffinity capture, charge neutralization-based polymer precipitation, size-exclusion chromatograph, and microfluidic techniques (124). Nevertheless, almost all technologies are more or less faced with the problems of a long time, low yield, high cost, and need to consider both purification rate and labor cost. Regarding exosome therapy, researchers will also pay attention to the source cell, quality control, and safety. Stable particle size, concentration, loading capacity, and the elimination of infectious particles are essential to ensure product homogeneity. In addition, there still needs to be clearer and unified standards or procedures for the transport, storage conditions, administration methods, and response to adverse reactions. The vigorous development of exosomes in the past 40 years has brought the dawn of precision treatment for many diseases. We urgently need to seize the opportunities, meet the challenges, and make efforts for clinical application as soon as possible.

## 5 Conclusions

Exosomes are important vectors for virus transmission and play an essential role in promoting viral infection and antiviral immunity. Its comprehensive source of cells and a variety of goods endow the exosomes with heterogeneity, providing plenty of biological functions and space for artificial transformation. With the development of isolation and purification technology, researchers’ focus has shifted from basic research to clinical application, especially vaccine development. We have reason to believe that exosomes will establish a unique research system in the viral diagnosis and treatment.

## Author contributions

All authors contributed to the article and approved the submitted version.
